# The effects of using multi-species probiotics in late-pregnant and lactating sows on milk quality and quantity, fecal microflora, and performance of their offspring

**DOI:** 10.14202/vetworld.2023.2055-2062

**Published:** 2023-10-07

**Authors:** Narathon Innamma, Natharin Ngamwongsatit, Kampon Kaeoket

**Affiliations:** 1 Department of Clinical Sciences and Public Health, Faculty of Veterinary Science, Mahidol University, Nakhon Pathom, 73170, Thailand; 2 Laboratory of Bacteria, Veterinary Diagnostic Center, Faculty of Veterinary Science, Mahidol University, Nakhon Pathom, 73170, Thailand

**Keywords:** fecal microflora, lactation, probiotics, swine, weaning

## Abstract

**Background and Aim:**

The dietary probiotics in sows during gestation to lactation period have gained considerable attention with respect to their beneficial effects on sows and their piglets’ performance and health. This study aimed to evaluate the effects of using probiotics in late-pregnant and lactating sows on milk quality, quantity, fecal microflora of sows, and growth performance of their offspring until weaning.

**Materials and Methods:**

Thirty-four sows were equally divided into two groups (control and treatment). Only those in the treatment group were fed 5 g of probiotics at 12 weeks of pregnancy, once daily for 7 weeks, until their piglets were weaned. Colostrum samples were collected at 3, 6, 12, and 24 h after farrowing and measured for immunoglobulin concentration. Percentages of fat, protein, and lactose in colostrum, colostrum production, total intake of immunoglobulin A (IgA), immunoglobulin G (IgG), fat, protein, and lactose, the change of fecal microflora of sows, and average daily gain of piglets were measured.

**Results:**

The results showed that there were no significant differences in the concentrations of IgA, IgG, and IgM in colostrum and the percentages of fat, protein, lactose, solid-not-fat, and total solid in colostrum between the groups; however, the colostrum production at 24 h in the treatment group (6,075.29 mL) was higher than in the control group (4,809.54 mL). Higher total intakes of IgA and IgG as well as total intake of fat, protein, and lactose, particularly at 3 h after farrowing, were found in the treatment group. Probiotic supplementation remarkably altered the microbiota community at the phylum level. We found that Firmicutes and Bacteroidetes are the dominant phyla, present in the gut of more than 90% of pregnant and lactating sows. Changes in microbial proportions were observed due to the changes of pig production stage. The weaning weight of the treatment group was higher than in the control group (6.34 ± 1.71 vs. 4.84 ± 1.29 kg, respectively).

**Conclusion:**

Feeding of multi-species probiotic BACTOSAC-P™ during late pregnancy and lactation in sows positively influenced colostrum production. In this experiment, the use of BACTOSAC-P™ improved the yield of colostrum production. The high immunoglobulin concentration and high yield of the colostrum of sows with a diet supplemented with BACTOSAC-P™ significantly reduced piglet mortality during the suckling period. Furthermore, the probiotic diet induced changes in the fecal microbial population in sows by increasing the number of microorganisms from the Firmicutes phylum, which had positive effects on sow health and their piglets, leading to better piglet growth performance.

## Introduction

Pigs are one of the major forms of livestock in Asian countries, including Thailand. They are raised in intensive housing environments, making them more susceptible to infectious diseases. Antibiotics are extensively used in pigs as growth promoters and preventive medicines. However, the increased use of antibiotics leads to an increase in antibiotic-resistant bacterial strains, which can harm both humans and animals [1]. It has been suggested that a colistin-resistance gene (*mcr*-1) found in *Escherichia coli* originating from pigs may be transferred directly or indirectly to humans [2]. In pig-producing countries globally, including Thailand, increasing attention is being paid to reducing the use of antibiotics for preventive purposes, with only clinical application for treating sick animals being allowed. Unfortunately, this reduced use of antibiotics has caused an increase in bacterial infection in pigs. The associated morbidity and mortality rates in the weaning period have been reported to be high [3]. Therefore, the replacement of antibiotics with an alternative to reduce the losses of pigs and not facilitate antibiotic resistance has been pursued. Such alternatives to replace antimicrobials include antibacterial vaccines, immunomodulatory agents, bacteriophages, antimicrobial peptides, probiotics, prebiotics, synbiotics, plant extracts, and feed enzymes [4].

Probiotics are living microorganisms that promote host gut health. They confer benefits such as maintaining homeostasis in the gastrointestinal (GI) tract, and improving digestion and the overall health of the host [5]. In the last decade, many studies have reported on the use of probiotics in pigs, for example, using *Lactobacillus amylovorus* and *Enterococcus faecium*, *Lactobacillus reuteri, Bacillus licheniformis*, and *Bacillus subtilis* to establish good gut health [6–8]. It has also been recently reported that some probiotics showed an inhibitory effect on pig bacterial pathogens [9, 10], subsequently leading to higher average daily gain (ADG), lower feed conversion ratio (FCR), and improved physiological conditions [11]. Probiotics in the GI tract also inhibits colonization by other pathogens, preventing them from establishing themselves in the animal’s body. Moreover, lactic acid-producing bacteria (LAB), such as *Lactobacillus* spp., can create acidic environments unsuitable for particular pathogens [9]. Besides, probiotics can also stimulate the host immune system by modulating toll-like receptors (TLRs) on cell membranes of the GI tract and signaling the production of immunostimulant cytokines [12]. In newborn piglets, colostrum, which is high in immunoglobulin G (IgG) and immunoglobulin A (IgA) content, can promote health, growth, intestinal growth and function, and immunity of piglets [13–15]. It can also provide sufficient energy for thermoregulation during the first 24 h of life, which is critical for the survival of piglets. It was reported that piglets with colostrum consumption of 250 g could achieve good health and growth [16, 17]. The gut microbiota composition of sows during pregnancy and lactation can impact enteric nutrient absorption and immunity [18], which influences the body weight (BW) of piglets at birth and weaning, the number of piglets born alive, the rate of pre-weaning mortality, and the number of piglets weaned per litter [19]. It has been documented that adding a single strain of probiotics (*B. licheniformis* or *B. subtilis* spores) in pig feed could increase intake during lactation in sows, prevent body mass loss, improve colostrum content (i.e., immunoglobulin content, fat, protein, lactose, solid-not-fat [SNF], and total solid [TS]), decrease the occurrence of diarrhea in piglets, lower the pre-weaning mortality rate, and increase the weaning weight [20]. Nonetheless, little information has been reported on the effects of multi-species probiotics on quantity and quality of the colostrum of sows and the performance of their offspring.

Therefore, we studied the effects of using a multi-species probiotic product in late-pregnant and lactating sows on milk quality and quantity, the changes in fecal microflora of sows, and the performance of their offspring.

## Materials and Methods

### Ethical approval

This research project was approved by the Faculty of Veterinary Science, Mahidol University-Institute Animal Care and Use Committee (MUVS-2018-06-26).

### Study period and location

This study was conducted from October 2018 to December 2019 on a private farrow-to-finish pig farm with an open house system in Chonburi province, Thailand. The farm was with approximately 3,000 sows.

### Probiotics

The multi-species probiotic product (BACTOSAC-P™; K.M.P. Biotech Co., Ltd., Thailand) used in this study is composed of seven living strains of different bacteria at different concentrations (Table-1). According to a molecular test performed at the Department of Microbiology, Faculty of Medicine Siriraj Hospital, Mahidol University, none of these probiotic strains carried an antibiotic resistance gene (unpublished data). The recommended dose of these probiotics was 5 g per meal sprinkled on top of the feed, once daily from week 12 of pregnancy until weaning of sows, for 7 weeks in total.

**Table-1:** Composition of multi-species probiotic product (BACTOSAC-P™).

	Microbial composition	Content per gram of product
1.	*Lactobacillus acidophilus*	1.0×10^7^CFU/g
2.	*Lactobacillus plantarum*	1.0×10^7^CFU/g
3.	*Enterococcus faecium*	1.0×10^7^CFU/g
4.	*Pediococcus pentosaceus*	1.0×10^6^CFU/g
5.	*Bacillus subtilis*	1.0×10^7^CFU/g
6.	*Bacillus licheniformis*	1.0×10^7^CFU/g
7.	*Saccharomyces cerevisiae*	1.0×10^6^CFU/g

CFU=Colony-forming unit

### Experimental animals

Overall, 34 Landrace × Yorkshire primiparous and multiparous sows (at 12 weeks of pregnancy) were randomly divided into two groups: Control group (n = 17 sows) and treatment group (n = 17 sows, fed with BACTOSAC-P™ powder). They were kept in an open-housing system, fed with farm mixed feed at 5–6 kg/day, and provided with water *ad libitum*. In the treatment group, sows were fed a standard diet supplemented with probiotic at 5 g/day starting on day 84 of gestation until the end of the 24-day lactation period. Meanwhile, in the control group, sows were fed a standard diet for late gestation and lactating sows until the end of the 24-day lactation period.

### Data collection

At least 16 mL of sow colostrum was collected in both groups by hand milking [21] at 3, 6, 12, and 24 h after farrowing from four to six teats in the anterior part of the udder. Colostrum samples were kept at 4°C during transportation to the laboratory and stored at −20^o^C until further analysis of milk composition and immunoglobulins.

Before analysis, the frozen samples were thawed at room temperature (28°C–30°C). Three milliliters of colostrum was used to measure the level of immunoglobulin using Pig Ig enzyme-linked immunoassay test kits, in accordance with the manufacturer’s instructions (Koma Biotech, Seoul, Republic of Korea). Thirteen milliliters of colostrum was used to analyze milk composition (i.e., % fat, % protein, % lactose, SNF, and TS) by Milkoscan™ FT1 (Foss Electric, Hillerød, Denmark) [22, 23].

### Sow colostrum production

Sow colostrum production was indirectly calculated from the piglet BW gain at 24 h after farrowing and transformed into the colostrum intake (CI) using the following equation of Theil *et al*. [24]:

CI (g) = −106 + 2.26 WG + 200 BWB + 0.111D − 1,414 WG/D + 0.0182 WG/BWB

Where CI = Colostrum intake (g), WI = 24 h weight gain of piglet (g), BWB = Pig body weight at birth (kg), and D = Time elapsed from birth to weighing at t_24_ (min).

### Calculation of total intake of IgA, IgG, protein, fat, and lactose

The total intake of IgA and IgG at 3, 6, and 12 h was calculated as follows:

Total intake of Ig = concentration of Ig × (colostrum production at 24 h/average number of piglets born alive in each group).

The total protein, fat, and lactose intake was also calculated using the same equation.

### Growth performance of piglets

The ADG of piglets in each group was calculated as follows: ADG = (BW_wean_ − BW_birth_)/Day

Where ADG = Average daily gain (g/day), BW_wean_ = Pig body weight at weaning (kg), BW_birth_ = Pig body weight at birth (kg), and Day = Nursing period (day).

### Microbiota diversity analysis

Pooled samples of feces were collected from sows in both groups at week 12 of pregnancy and at 1 week after farrowing. Total DNA was extracted from fecal samples using QIAamp Stool DNA Extraction kit (QIAGEN, Germany). 16s rRNA was amplified from each pooled DNA sample and then sequenced using the Illumina MiSeq platform (San Diego, CA, USA). Overlapping paired-end reads were assembled using PEAR. The reads were processed using Quantitative Insights into Microbial Ecology v1.9.0 [25] for operational taxonomic units picking and taxonomy.

### Statistical analysis

Data on colostrum quality and quantity, and performance of the offspring were analyzed using the general linear model from IBM Statistical Package for the Social Sciences Software version 22.0 (IBM Corporation, New York, USA). The t-test was employed to compare the volume of colostrum and composition (i.e., fat, protein, lactose, SNF, and TS), piglet performance, and immunoglobulin content (i.e., IgA, IgM, and IgG). All data are presented as mean ± standard deviation and the differences between the results of the treatment and control groups were considered statistically significant at p ≤ 0.05.

## Results

### Milk quality and quantity

The immunoglobulin levels (i.e., pig IgG, IgA, and IgM) and colostrum composition in the control and treatment groups are shown in Tables-1 and 2, respectively. The two groups had no significant difference in the immunoglobulin levels (p > 0.05). The IgA level in the treatment group peaked at 12 h (17.46 ± 5.30 mg/mL), while the peak in the control group occurred at 6 h (20.51 ± 6.20 mg/mL). Regarding the IgG level in the control and treatment groups, their peaks were found at 3 h post-farrowing (198.27 ± 79.66 vs. 179.74 ± 62.07 mg/mL) and gradually decreased over time. Unlike IgA and IgG levels, the IgM levels were lower than 10 mg/mL over time. In the milk composition analysis using Milkoscan™ FT1, there were no significant differences in terms of percentages of fat, protein, lactose, SNF, and TS. Fat and protein percentages gradually decreased over time, while the percentage of lactose tended to plateau.

**Table-2:** Concentration of immunoglobulin (mg/mL) in colostrum at 3, 6, and 12 h after farrowing in control and treatment groups.

Parameters	Control	Treatment	p-value
IgG			
3 h	198.27 ± 79.66	179.74 ± 62.07	0.91
6 h	189.40 ± 77.08	164.85 ± 90.42	0.71
12 h	158.59 ± 26.85	163.61 ± 121.08	0.52
IgA			
3 h	18.62 ± 5.16	16.36 ± 6.97	0.93
6 h	20.51 ± 6.20	16.87 ± 6.95	0.36
12 h	17.91 ± 4.78	17.46 ± 5.30	0.91
IgM			
3 h	4.75 ± 1.21	7.35 ± 3.30	0.33
6 h	5.11 ± 1.34	4.93 ± 1.27	0.89
12 h	4.42 ± 0.86	4.05 ± 1.27	0.94

IgG=Immunoglobulin G, IgA=Immunoglobulin A, IgM=Immunoglobulin M

### Sow colostrum production and piglet performance

The calculated sow colostrum production revealed higher colostrum production in the treatment group than in the control group (6,075.29 ± 1,419.46 g/L and 4,809.54 ± 616.49 g/L). Therefore, we calculated the average piglet CI at 24 h using the number of piglets born alive in both treatment (13.5 piglets/L) and control (14.0 piglets/L) groups. The results revealed a higher CI in the treatment group than in the control group (450.02 ± 105.15 g/piglet vs. 343.54 ± 44.04 g/piglet). This resulted in a higher total intake of IgA, IgG, fat, protein, and lactose by each piglets at 3, 6, and 12 h in the treatment group than in the control group, as shown in Tables-3 and Table-4. Regarding the growth performance of piglets, ADG was significantly higher in the treatment group (176.0 ± 63.22 g/day; n = 201) than in the control group (121.0 ± 47.23 g/day; n = 209) (p < 0.05). On comparing the pre-weaning mortality rate, this rate was lower in the treatment group (10.95%) than in the control group (14.83%).

**Table-3:** Percentages of fat, protein, lactose, SNF and TS in colostrum at 3, 6, and 12 h after farrowing in control and treatment groups.

Colostrum composition	Control	Treatment	p-value
Fat			
3 h	7.94 ± 0.73	7.56 ± 1.25	0.66
6 h	9.51 ± 1.48	6.53 ± 1.97	0.00
12 h	7.88 ± 1.34	5.82 ± 1.82	NA
Protein			
3 h	13.28 ± 0.49	15.51 ± 2.42	0.18
6 h	15.43 ± 1.97	14.91 ± 1.97	0.71
12 h	14.95 ± 1.67	13.59 ± 2.30	NA
Lactose			
3 h	3.32 ± 0.40	2.98 ± 0.40	0.29
6 h	2.81 ± 0.67	2.99 ± 0.39	0.59
12 h	3.73 ± 0.61	3.39 ± 0.43	NA
SNF			
3 h	16.74 ± 0.11	18.40 ± 1.81	0.19
6 h	18.2 ± 1.27	17.87 ± 1.46	0.75
12 h	18.55 ± 1.14	17.06 ± 1.72	NA
TS			
3 h	26.09 ± 0.71	27.63 ± 2.98	0.43
6 h	29.42 ± 2.96	26.06 ± 2.94	0.13
12 h	28.08 ± 2.52	24.36 ± 3.16	NA

NA=Not analyses, SNF=Solid not fat, TS=Total solid

**Table-4:** The total intake by calculation of fat, protein, lactose, SNF, and TS by piglets during 12 h in control and treatment groups.

Item	Hours	Control	Treatment
Colostrum intake (g/piglet)		343.54	405.02
Total IgG intake (mg/piglet)	3 h	68,113.68	72,798.30
	6 h	65,066.48	66,767.55
	12 h	54,482.01	66,265.32
Total IgA intake (mg/piglet)	3 h	6,396.72	6,626.13
	6 h	7,046.01	6,832.69
	12 h	6,152.80	7,071.65
Fat (g/piglet)	3 h	27.28	30.62
	6 h	32.67	26.45
	12 h	27.07	23.57
Protein (g/piglet)	3 h	45.62	62.82
	6 h	53.01	60.39
	12 h	51.36	55.04
Lactose (g/piglet)	3 h	11.41	12.07
	6 h	9.65	12.11
	12 h	12.81	13.73

SNF=Solid not fat, TS=Total solid, IgG=Immunoglobulin G, IgA=Immunoglobulin A

### Changes of sow fecal microbial diversity

**Figure-1:**
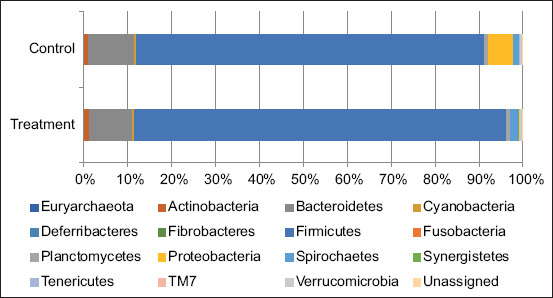
Distribution of the phylum as a percentage of the total number of identified 16s rRNA sequences from fecal samples of the 12^th^ week pregnant sows.

**Figure-2:**
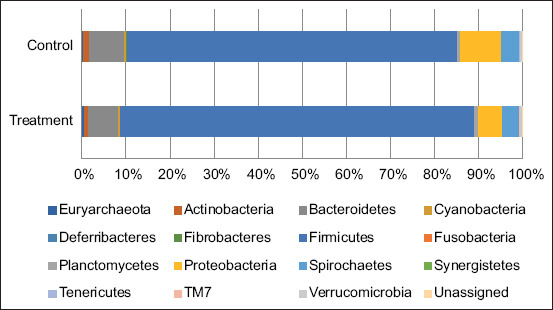
Distribution of the phylum as a percentage of the total number of identified 16s rRNA sequences from fecal samples of the 1^st^ week lactating sows.

The results of genomic (DNA) sequencing at the phylum level are presented in Figure-1. A total of 16 phyla were found in the fecal samples of the sows in the 12^th^ week of pregnancy. At the phylum level, Firmicutes and Bacteroidetes were dominant taxa of more than 80% of the total sequences when compared with other phylum, Firmicutes for more than 79.32% and 80.94%, and Bacteroidetes for approximately 10.59% and 9.29% in control and treatment groups, respectively. As shown in Figure-2, the percentages of dominant phyla of gut microbes at 1 week of lactation in pregnant sows in the control group were as follows: 75.04% for Firmicutes, 9.39% for Proteobacteria, 7.88% for Bacteroides, and 4.14% for Spirochaetes. Meanwhile, in the treatment group, the proportions were 80.25% for Firmicutes, 6.62% for Bacteroides, 5.51% for Proteobacteria, and 3.83% for Spirochaetes. The microbial diversity analysis showed shifts in the gut microbial diversity only in sows in the control group. The results showed that the levels of Firmicutes and Bacteroidetes decreased during the lactation period.

## Discussion

Taking the findings together, it was suggested that feeding pregnant sows with multi-species probiotics from week 12 of pregnancy over a 7-week-period benefits milk quality and quantity and the growth performance and pre-weaning mortality of their offspring. Many mechanisms can be proposed to explain the obtained results. For example, providing probiotics has been proven in many studies to improve the balance in gut microbiota, the production of volatile fatty acids and vitamins, and the development of the immune system [26]. Besides, it has been demonstrated that probiotic feeding can increase feed consumption and nutrient utilization in sows during pregnancy and lactation [20]. This may explain the superior colostrum production of sows in the treatment group in this study.

It is well documented that probiotics contain various microbiota strains and benefit the animal’s overall health. Recently, *in vitro* and *in vivo* findings have been reported describing that LAB and *Bacillus* spp. can inhibit the growth of pathogenic bacteria from pigs [9, 10] and improve gut health and performance of nursing and finishing pigs in an experimental study [20]. Particular *Lactobacillus* species have the ability to prevent the colonization of pathogens through competitive exclusion and promotion of the production of short-chain fatty acids (acetic acid and propionic acid), which can suppress pathogens by creating an acidic environment [27]. They also enable nutrients that are not normally digestible by the host to be digested and absorbed, hence helping to improve the health status and growth of the weaned pigs [28]. *Lactobacillus* and *Pediococcus* strains can enhance the antioxidative defense system of weanling pigs and consequently prevent intestinal infections caused by enterotoxigenic *E. coli*. Bacillus probiotics positively affect pigs, such as higher weight gain, improved FCRs, and reduced incidence of diarrhea and mortality in piglets [29]. This may support the present results in that feeding on multi-strain probiotics could regulate gut health by modulating bacterial communities in the intestinal tract, improving feed efficiency and growth rate, enhancing the production of short-chain fatty acids, and inhibiting pathogens in the animals [28]. However, it is worth noting that the effects of probiotics could vary depending on the particular strains of microbiota contained within them. The multi-strain probiotics used in this study contained *Lactobacillus* spp., *Pediococcus* spp., *Bacillus* spp., *Enterococcus* spp., and *Saccharomyces* spp., which could have multiple functions and benefits for all of the sows and their piglets.

A previous study by Zhang *et al*. [8] in pigs showed that probiotics can modulate the immune system by regulating immune cell activation and increasing antibody production. Toll-like receptors are among the key recognition receptors in the innate immune system. Increased TLR expression releases pro- and anti-inflammatory cytokines when probiotics are used to stimulate the innate immune system. It has also been reported that *Bacillus amyloliquefaciens* SC06 alleviated the intestinal inflammation of fattening pigs by regulating the expression of proinflammatory cytokines, including interleukin (IL)-6, IL-8, and monocyte chemotactic protein 1 [30]. Besides *Bacillus*, *Lactobacillus fermentum* and *Pediococcus acidilactici* also reduced the concentration of the serum proinflammatory cytokines IL-6 and interferon-g in the serum of weaned piglets and helped to reduce the damage caused by inflammation [28]. Moreover, Laskowska *et al*. [31] showed high levels of pro- and anti-inflammatory cytokines in serum of pregnant sows, indicating that probiotic supplementation has an immunomodulatory effect on systemic immune processes, manifested in part as improvement in the immune quality of the colostrum. The use of multimicrobial probiotic formulation products as dietary supplements in sows increased the concentrations of the proinflammatory cytokines tumor necrosis factor-a and IL-6, which increased the protective capacity of the colostrum by stimulating immune cell mechanisms protecting the sows and their piglets against infection. Besides, the results from study of Laskowska *et al*. [31] showed increased concentrations of anti-inflammatory cytokines IL-4, IL-10, and transforming growth factor-β, as well as IgG and IgA in the colostrum and milk from sows in the experimental group. This indicated the immunoregulatory effect of multimicrobial probiotics on Th2 cells and increased regulatory T cell expression. These findings correspond to the results of previous studies by Laskowska *et al*. [31], Jarosz *et al*. [32], and Tsukahara *et al*. [33], which show that including probiotics in animal diets during pregnancy and lactation increased the immune potential of colostrum and milk and protected against infectious diseases. In this study, although there was no significant difference in the IgG and IgA concentrations in sow colostrum between the groups, the greater production of colostrum and milk by sows in the probiotic-supplemented group resulted in a higher intake of colostrum by the piglets during the first 24 h of life. The piglets of the sows given probiotics consumed more colostrum at 24 h after farrowing (309.3 g/piglet), so they consumed more total fat, total protein, total lactose, and total immunoglobulins (i.e., IgA and IgG), resulting in higher growth performance and lower pre-weaning mortality than in the control group. This is in agreement with the previous studies by Nuntapaitoon *et al*. [16] and Nuntapaitoon *et al*. [17], in which it was proposed to achieve good health in pre- and post-weaning piglets, 250 g of colostrum should be consumed in the first 24 h after birth, with a minimum of 200 g/piglet. Furthermore, positive correlations have been identified between increased CI and decreased mortality rate, and a subsequent long-term increase in piglet growth [32]. This would have been due to the higher levels of immunoglobulin, fat, protein, and energy transferred to the piglets, which are essential for piglet survival [20, 32–36]. The use of BACTOSAC-P™ during pregnancy and lactation positively affected growth performance and piglet health. The high level of immunoglobulins in colostrum of the sows whose daily diets were supplemented with BACTOSAC-P™ significantly reduced piglet mortality. This might be explained by the probiotics exerting immunomodulatory effects, increasing secretory IgA and secretory IgG in colostrum/milk, which, in turn, prevented infection by pathogenic bacteria in the GI tract of piglets. This would subsequently influence piglet performance and reduce pre-weaning mortality at this particular pig farm. In agreement with the present results, the piglets in the treatment group showed significantly higher ADG than the piglets in the control group, which was attributed to the higher amount of colostrum consumed, so more passive immunity was received and better growth subsequently occurred [31, 32, 35].

The ecosystem of the GI tract is complex and plays an important role in both promoting health and preventing disease. A number of studies have investigated the metagenomic characteristics of the intestinal microbiota after probiotic supplementation in pig [37–39]. An increased level of LAB was mainly observed in the analysis of the microbiota of suckling piglets after supplementation with *Lactobacillus* probiotics. The microbiome of the intestinal tract of pig undergoes a post-weaning transition where lactobacilli dominate the intestinal microbiota of suckling piglets, while members of Firmicutes and *Bacteroides* are predominant in adult pigs [38, 39]. During the weaning process, lactobacilli populations were detected at significantly lower levels after exposure to stress factors, suboptimal feed intake, and transportation [39]. Furthermore, a shift in composition and activities of the predominant microbiota, and emergence of clostridia and *E. coli*, were encountered in the intestinal tract of the piglets early in the post-weaning stage [40]. However, it has been indicated that the supplementation of probiotics can reduce the number of pathogenic bacteria in the gut while increasing the relative abundance of commensal lactobacilli and bifidobacteria [41]. Many studies have shown that probiotic supplementation could help to balance the bacterial community [39–41], which may also promote the health of sows. In agreement with our study, probiotic supplementation has been reported to induce remarkable alterations in the microbiota community at the phylum level. We found that Firmicutes and Bacteroidetes are the dominant phyla present in the gut of more than 90% of pregnant and lactating sows. Changes in microbial proportions were observed in this study, which might be due to the changes of pig production stage. The ratio of these two phyla in the digestive tract can affect the capacity to absorb nutrients from ingested feed. It has also been reported that body fat percentage is positively correlated with the abundance of Firmicutes in the gut microbiota [42]. Cui *et al*. [43] demonstrated that *B. subtilis* probiotics decreased the number and percentage of *Bacteroides* and increased the percentage of Firmicutes in cecal contents. Furthermore, pigs receiving probiotic supplementation had higher backfat thickness than control pigs. Backfat thickness is one of the significant parameters of female pigs correlated with reproductive performance, for example, puberty attainment, total piglets born, and farrowing rate. Besides, backfat is one of the significant sources of hormones related to puberty attainment, such as leptin, insulin-like growth factor-I, and progesterone (P4) [44].

## Conclusion

The feeding of sows on multi-species probiotics (BACTOSAC-P™) during late pregnancy and lactation positively affected colostrum production. In this experiment, the use of BACTOSAC-P™ improved colostrum yield in sows by improving the digestibility of dietary nutrients and overall feed efficiency owing to the production and activity of digestive enzymes by *Bacillus* spp. contained in the probiotic. Furthermore, the probiotic type used in this study is composed of a variety of microbial probiotics, for example, *Lactobacillus* spp., *Pediococcus* spp., *Enterococcus* spp., *Bacillus* spp., and *Saccharomyces* spp., which have multiple functions in promoting colostrum production and immunoglobulin production in colostrum/milk, reducing piglet mortality, achieving better growth performance results, and reducing financial losses of farms.

## Authors’ Contributions

KK: Study conception and design. NI, NN, and KK: Conducted experiments and wrote the manuscript. NI and KK: Analyzed data. All authors have read, reviewed, and approved the manuscript.
